# Intelligent Sensing for Transportation Safety: Technologies, Innovations, and Challenges

**DOI:** 10.3390/s26144438

**Published:** 2026-07-13

**Authors:** Pengcheng Wang, Yang Yang, Guangnian Xiao

**Affiliations:** 1 School of Astronautics, Beihang University, Beijing 100191, China; pcwang@buaa.edu.cn; 2 School of Traffic and Transportation, Beijing Jiaotong University, Beijing 100044, China; bjtuyang@bjtu.edu.cn; 3 School of Economics and Management, Shanghai Maritime University, Shanghai 201306, China

## 1. Introduction

Transportation safety remains a critical global concern, with road traffic crashes causing approximately 1.2 million annual deaths and 20–50 million non-fatal injuries worldwide [1]. As the core “perceptual system” of intelligent transportation systems (ITS), advanced sensing technologies—combined with artificial intelligence (AI)—have become essential to mitigating these risks, enabling real-time monitoring of driver states, vehicle dynamics, and road environmental conditions [2,3]. However, the sector faces unprecedented challenges: on one hand, driver-related factors (e.g., fatigue, road hypnosis) account for 78% of traffic crashes [4], yet traditional sensing methods struggle to detect subtle physiological changes in dynamic, non-monotonous driving environments. On the other hand, the integration of deep learning into key perception modules (e.g., traffic sign detectors, pedestrian recognizers) has exposed vulnerabilities to backdoor attacks, where malicious triggers can manipulate model outputs without compromising normal performance—posing severe threats to safety-critical applications [5,6]. Concurrently, the rapid growth of advanced driving assistance systems (ADAS) and autonomous vehicles (AVs) has intensified demand for reliable, robust sensing solutions that can bridge laboratory research and real-world deployment [7].

This Special Issue focuses on Intelligent Sensing for Transportation Safety, compiling eight high-quality research papers that address key bottlenecks in the field from diverse perspectives: driver state identification, short-term driving risk prediction, traffic sign detection and quality assessment, and perception system security. These studies not only propose innovative technical frameworks but also validate their effectiveness through real-vehicle experiments and large-scale dataset testing, providing scientific support for the transformation of transportation safety technologies. This Special Issue includes eight research papers (the core focus of this editorial). The following sections will overview the key contributions of each paper, rather than elaborating on each work in detail—we encourage readers to explore these scholarly works firsthand for deeper insights.

## 2. An Overview of Published Articles

Longfei Chen’s article (Contribution 2) addresses the long-standing challenge of road hypnosis identification in non-fixed driving scenarios—a critical gap in existing research that has historically focused on monotonous highways. Road hypnosis, an unconscious driving state induced by environmental monotony and driver physiological fatigue, impairs reaction speed and environmental perception but is difficult to detect in dynamic urban settings.

The study recruited 30 participants with ≥8 years of driving experience (to avoid novice driver bias), collecting data during their daily commutes (covering urban roads, CBD business districts, and school zones) to simulate real-world variability. A multi-source sensing system captured three categories of data: physiological data (eye movement parameters via aSee Glasses eye-tracking glasses and EEG signals via Enobio Dx device), vehicle data (speed, acceleration, and jerk via AutoNavi-integrated platform), and environmental data (road type and traffic density via dashcam). Feature extraction tailored to each data type—power spectral density (PSD) for EEG, local linear embedding (LLE) for eye movement, sliding window for vehicle dynamics—was integrated into an XGBoost-HMM hybrid model. This model combines XGBoost’s strength in nonlinear feature learning and HMM’s capacity to model temporal state transitions, achieving 94.9% identification accuracy (5-fold cross-validation) and outperforming traditional HMM (88.3%) and random forest (92.66%). Feature importance analysis via SHAP and LIME identified beta waves (EEG), delta waves (EEG), right pupil diameter, and vehicle speed as top discriminative indicators—beta wave intensity decreased by 35% (reduced alertness) and delta wave intensity increased by 28% (enhanced drowsiness) during road hypnosis. The study provides a technical foundation for integrating physiological-vehicle data into intelligent cockpits, supporting multi-modal warnings to restore driver attention.

Xinpeng Yao’s article (Contribution 7) focuses on short-term driving risk prediction—a core function of ADAS that enables early collision warnings—addressing two key limitations of existing research: ambiguous risk thresholds and suboptimal temporal parameters.

First, the authors calibrated critical risk indicators via Monte Carlo simulation (10,000 iterations) to align with real-world dynamics: the reciprocal of time-to-collision (TTCi) threshold was set to 0.86 s^−1^ (simulating front-vehicle random deceleration via gamma distribution and driver reaction time via lognormal distribution), and the braking deceleration threshold to −2.6 m/s^2^ (derived from rear-vehicle maximum braking capacity via truncated normal distribution). A variable sliding window approach optimized temporal parameters, evaluating F1-scores across 25 combinations of prediction lead time (1–6 s) and window length (1–6 s)—the optimal configuration (1.6 s lead time, 1.2 s window length) balanced early warning adequacy and prediction accuracy.

The framework also included Boruta-based feature selection (filtering 39 key features from 98 initial variables, reducing complexity by 60%) and random undersampling to address class imbalance (942 risk vs. 3533 normal samples). Among four tested models (logistic regression, multilayer perceptron, XGBoost, Light Gradient Boosting Machine (LGBM)), the LGBM achieved the best performance: recall = 90.2%, precision = 85.71%, F1 = 87.9%. Notably, fusing human-vehicle-road-environment (HVRE) data significantly improved accuracy—fusion achieved 87.9% F1, compared to 79.59% (driver manipulation data alone), 79.13% (vehicle attitude data alone), and 81.22% (surroundings data alone). Pilot testing in Shandong Hi-Speed Group’s ADAS reduced false alarms by 12% and increased early warning lead time by 1.6 s, providing a practical solution for preventing rear-end collisions in stop-and-go traffic.

Yaojung Shiao’s article (Contribution 6) addresses a highly specific yet common safety hazard for vulnerable road users: collisions between motorcycles and suddenly opened car doors. Such crashes are particularly relevant in dense urban traffic, where roadside parking and mixed motorcycle–car interactions create limited reaction time for riders. To provide a low-cost alternative to radar-based systems, the study integrates a camera-based deep learning detection module with an automatic braking assistance strategy for motorcycles.

The authors first developed a dataset for car-door state recognition, classifying door conditions into closed, small opening, medium opening, and large opening states. Among several YOLOv12 variants, the YOLOv12s model achieved the best overall balance between accuracy and real-time feasibility, with precision, recall, and mAP@0.5 values of 90.5%, 80.6%, and 87.8%, respectively. The detected door state was then combined with time-to-collision, rider braking action, and initial braking speed to determine braking intensity levels of 0%, 25%, 50%, or 100%. In parallel, the study used BikeSim-based simulations to optimize the front braking ratio according to vehicle speed. At an initial braking speed of 60 km/h, a 45% front braking ratio reduced braking stroke by 35.61% and 13.37% compared with 20% and 60% front braking ratios, respectively. With an estimated total system latency of approximately 0.5 s, the proposed framework demonstrates the feasibility of combining intelligent perception and adaptive braking control to prevent motorcycle–car door collisions.

Yu Miao’s article (Contribution 8) extends intelligent transportation sensing to underground autonomous driving, focusing on navigable area computation for trackless rubber-wheeled vehicles in coal mine tunnels. Unlike open-road environments, underground tunnels suffer from weak illumination, water mist, dust, uneven ground, dynamic width changes, intersections, and shunting chambers, all of which degrade conventional image-based or open-road LiDAR boundary detection methods.

To address these challenges, the study proposes a rule-based LiDAR framework centered on ground feature extraction and boundary processing. The method first performs real-time point cloud correction through pre-correction and dynamic updating to align ground point clouds with the LiDAR coordinate system. Corrected point clouds are then projected into a two-dimensional grid map to reduce the influence of uneven terrain on boundary extraction. For complex tunnel structures, an adaptive boundary completion method is introduced to recover discontinuous boundaries in intersections and shunting chambers. Finally, temporal context is fused to maintain continuous boundary extraction during vehicle operation. Experiments using real underground vehicle data across straight tunnels, curved tunnels, intersections, and shunting chambers showed precisions of 97.5%, 93.2%, 85.0%, and 88.3%, respectively, with an average processing time of 35 ms per frame. The study provides a practical real-time safety redundancy for underground autonomous transportation, especially in harsh environments where deep learning perception may become unreliable.

Ziyad N. Aldoski’s article (Contribution 1) evaluates the performance of the YOLOv8 algorithm in traffic sign detection (TSD) and quality assessment, addressing the gap between algorithmic performance and real-world sign material properties. Traffic signs are critical for guiding driver behavior, but their visibility is heavily influenced by lighting and retroreflective material degradation—factors often overlooked in pure algorithmic studies.

The study constructed the ZND dataset (16,500 labeled images) for scenario diversity: sourced from the German Traffic Sign Recognition Benchmark (GTSRB) for standard signs, GitHub repositories for rare signs (e.g., “Bicycle Pass”), and real-world photographs from Győr, Hungary (covering daytime, nighttime, and overcast conditions). To link detection performance with physical quality, a RetroSign GRX 554 handheld retroreflectometer (complying with EN 12899-1 [8]) measured the retroreflectivity coefficient (RA) of 190 signs, classifying them into RA1 (mean RA = 29 cd·lx^−1^·m^−2^, aging signs) and RA2 (mean RA = 187 cd·lx^−1^·m^−2^, new signs).

The YOLOv8 model was trained with optimized parameters (input size = 640 × 640, learning rate = 0.01, batch size = 16, 50 epochs) on a Kaggle NVIDIA A100 GPU. Key results included daytime and nighttime detection rates of 0.82 and 0.83 (no significant difference via independent *t*-test, *p* = 0.736), attributed to YOLOv8’s low-light feature extraction and synthetic data augmentation; RA2 signs exhibited 9% higher nighttime detection stability (relative standard deviation = 9% vs. 23% for RA1), with RA > 100 cd·lx^−1^·m^−2^ identified as the “critical threshold” for reliable nighttime detection; sharpness (Laplacian variance) and overall intensity (average pixel value) strongly correlated with detection rates—nighttime sharpness (1452) was 82% lower than daytime (8071), leading to 10% more misdetections. Human evaluations (61 participants) revealed a weak correlation between human perception and algorithmic detection (Pearson r = 0.42), providing guidance for transportation departments to prioritize RA2 materials and regular retroreflectivity inspections.

Shiwei Shao’s article (Contribution 5) broadens the scope of intelligent sensing from road-level perception to infrastructure-level monitoring and emergency management. Focusing on reservoir safety and sustainable operation, the study develops an integrated multi-sensor information system for real-time reservoir monitoring and management, which is highly relevant to the broader theme of sensing-supported safety governance for critical infrastructure.

The proposed framework adopts a four-layer architecture consisting of perception, data, model, and application layers. The perception layer integrates satellite, UAV, land-based, shipborne, and dam-body monitoring platforms to construct a three-dimensional sensing network. The data layer processes heterogeneous information through a “physical–semantic–application” correlation mechanism, enabling the fusion of structured sensor data, semi-structured reports, and unstructured images, videos, and point clouds. The model layer couples water safety, water resources, water environment, and water ecology models to support multi-objective decision-making. The application layer implements a closed-loop “forecasting–warning–simulation–planning” management process. The framework was validated at the Ye Fan Reservoir in Hubei Province, China, where GNSS receivers, seepage pressure gauges, water-level sensors, rainfall stations, video monitoring devices, termite monitoring equipment, and water quality sensors were integrated into a unified platform. After implementation, data delay was reduced from more than one hour to near real-time, flood forecasting error decreased from 20 to 30% to below 10%, emergency response time was shortened from two hours to ten minutes, and annual maintenance cost was reduced from RMB 500,000 to RMB 200,000. This work demonstrates how heterogeneous sensor networks and digital management platforms can enhance infrastructure resilience and operational safety.

Yueyue Chen’s article (Contribution 3) focuses on pedestrian flow modeling and emergency evacuation simulation, linking visual trajectory extraction with data-driven cellular automata. Pedestrian movement in public spaces such as overpasses, stations, and corridors is closely associated with congestion, evacuation efficiency, and crowd safety, yet many traditional simulation models rely on simplified assumptions rather than real behavioral data.

The study collected unidirectional pedestrian flow videos on an urban overpass under different density conditions, involving groups of 5, 10, 15, and 20 pedestrians. YOLO 11 and DeepSORT were used to extract and track pedestrian trajectories, while perspective transformation corrected image distortion and mapped pixel coordinates to physical space. Based on the extracted trajectories, the authors quantified pedestrian turning-angle distributions and converted them into data-driven transition probabilities for the Moore neighborhood in a cellular automaton model. The improved model further incorporated the speed–density relationship, panic coefficient, individual life value, group behavior, and hazard-source dynamics. Multi-scenario simulations covering normal evacuation, grouped movement, fire, and robbery scenarios revealed several important behavioral mechanisms: moderate panic may accelerate evacuation, whereas excessive panic causes disorder and unstable speed fluctuations; group movement is constrained by the slowest individual; and higher hazard-source speed reduces the proportion of safely evacuated pedestrians. This study provides a refined modeling framework for public-space evacuation analysis and shows how computer vision can directly inform pedestrian simulation rules.

Qiong Li’s article (Contribution 4) exposes a critical vulnerability in pedestrian detection systems, proposing the first backdoor attack using natural occlusions (e.g., backpacks, paper bags) as triggers—addressing the impracticality of existing attacks relying on unnatural digital perturbations or conspicuous patches. Pedestrian detectors are core to AV/ADAS safety, and their failure can directly lead to collisions.

The attack framework included three stealthy, physically deployable steps: trigger design (a heuristic algorithm generating natural occlusion regions, with “object-level embedding” placing triggers in the lower 2/3 of pedestrian bounding boxes at a 15–25% area ratio), poisoned sample generation (three embedding mechanisms: image-level, object-level, and image + object-level, with poisoned labels removing pedestrian bounding boxes), and model training/inference (validation on Faster R-CNN and RetinaNet using KITTI and CityPersons datasets, poisoning rates = 5–40%).

Key results included object-level embedding achieving average attack success rates (ASR) of 75.1% (KITTI) and 97.1% (CityPersons), while maintaining baseline benign performance (benign average precision = 42.2% vs. 42.5% for clean models); physical validation with a real backpack succeeded in indoor (2–8 m) and outdoor (2–8 m) scenarios, though the attack failed at 90° pedestrian rotation (ASR = 12%); and the attack maintained 68.3% ASR against test-time noise injection and 47.8% ASR against fine-tuning (a common defense), outperforming existing backdoor attacks (average 35% ASR under fine-tuning). The study highlights the need for occlusion anomaly detection and robust training to enhance pedestrian detector security, providing critical insights for AV safety design.

## 3. Conclusions

The eight research papers in this Special Issue deeply explore key issues in intelligent sensing for transportation safety—driver state identification, short-term risk prediction, traffic sign perception, and system security—from diverse perspectives. These findings not only help us comprehensively understand the challenges and opportunities facing ITS sensing technologies but also provide invaluable references for industry practitioners, policymakers, and researchers, laying a solid foundation for promoting transportation safety.

In technical practice, these studies have demonstrated tangible value: the road hypnosis identification model has been integrated into prototype intelligent cockpits, reducing driver inattention-related near-misses by 15%; the ADAS risk prediction framework has been piloted in commercial vehicles, improving rear-end collision warning accuracy by 12% [9]; the traffic sign quality assessment method has guided the replacement of 300+ low-retroreflectivity signs in Hungary, cutting nighttime misdetections by 20%; the backdoor attack research has prompted AV manufacturers to add “occlusion anomaly detection” modules, enhancing detector security by 40%.

The global ITS sector is currently undergoing a dual transformation of sensing performance enhancement and security resilience improvement. Recent studies have shown that multi-sensor fusion (e.g., LiDAR-camera-thermal imaging) can significantly improve extreme-scenario adaptability; federated learning and adversarial training are emerging as key to addressing backdoor vulnerabilities [10]; and low-power sensing technologies (e.g., solar-powered cameras) are advancing to support sustainable ITS deployment.

In future research, on the one hand, it is essential to further strengthen studies on the application of multi-sensor fusion and domain adaptation technologies in extreme scenarios, such as heavy fog, snow, and low-light environments, to enhance the adaptability and reliability of sensing systems across all real-world conditions; on the other hand, continuous attention should be paid to the balance between security and accuracy of perception models, developing lightweight backdoor defense mechanisms (e.g., pruning-based backdoor removal) that preserve detection performance while improving resilience, and avoiding the trade-off between security and practical usability. In addition, strengthening collaboration with standardization bodies such as ISO and IEEE to establish unified test protocols for backdoor resistance, retroreflectivity thresholds, and multi-modal fusion is also an important direction for future work, which can ensure consistency across research and industry and accelerate the translation of technical innovations to real-world applications. It is hoped that more researchers will join the study of intelligent sensing for transportation safety to jointly promote the continuous development and progress of this field. It is hoped that more researchers will join the study of intelligent sensing for transportation safety to jointly promote the continuous development and progress of this field.
